# Response of photosynthesis, the xanthophyll cycle, and wax in Japanese yew (*Taxus cuspidata* L.) seedlings and saplings under high light conditions

**DOI:** 10.7717/peerj.14757

**Published:** 2023-01-25

**Authors:** Wei Li, Jiacheng Li, Jia Wei, Chunda Niu, Deguang Yang, Baiwen Jiang

**Affiliations:** 1 Northeast Agricultural University, College of Resources and Environment, Harbin, Heilongjiang, China; 2 Northeast Agricultural University, College of Agriculture, Harbin, Heilongjiang, China; 3 Northeast Agricultural University, College of Horticulture and Landscape Architecture, Harbin, Heilongjiang, China

**Keywords:** Japanese yew, Photosynthesis, Fluorescence, Chlorophyll, Wax

## Abstract

In order to understand the adaptative changes of the Japanese yew (*Taxus cuspidate* L.) to high light conditions, this study investigated gas-exchange, chlorophyll fluorescence, chlorophyll, and the impact of epicuticular wax on the gas-exchange and photoinhibition of Japanese yew seedlings and saplings. The chlorophyll content per unit area and photosynthetic rate in seedling leaves were significantly lower than in sapling leaves. When leaves from seedlings and saplings were exposed to 1,200 μmol·m^−2^·s^−1^ photon flux density (PFD) for 2 h, seedling leaves exhibited a greater down-regulation of maximum quantum yield (Fv/Fm) and actual photosystem II efficiency ( $\Phi$PSII). Non-photochemical quenching (NPQ) and high energy quenching (qE) in sapling leaves were much higher than in seedling leaves when both were exposed to 1,200 μmol·m^−2^·s^−1^ PFD for 2 h. At a low level of O_2_, the photorespiration rate (P_r_) and the ratio of photorespiration/gross photosynthetic rate (P_r_/P_g_) in seedling leaves were lower than in sapling leaves when both were exposed to 1,200 μmol·m^−2^·s^−1^ PFD, but this difference did not reach statistical significance (*P* < 0.05). Compared with sapling leaves, seedling leaves exhibited lower levels of xanthophyll pool. Epicuticular wax content on seedling leaves was significantly lower than on sapling leaves. The results of this study showed that wax coverage on the leaf surface decreased the photosynthetic rate in sapling leaves as a consequence of decreased stomatal conductance. Epicuticular wax is related to tree age and photoinhibition prevention in the Japanese yew. It is possible that lower photosynthetic rate, lower NPQ depending on the xanthophyll cycle, and lower deposition of epicuticular wax results in seedling plants that are not adapted to high light conditions.

## Introduction

The Japanese yew (*Taxus cuspidata L*.) is an endangered and ornamental evergreen tree of the yew family (Taxaceae) (Wang et al., 2010) that grows in small, isolated populations in many parts of northern Asia (Kuang et al., 2019). The species has received attention because its bark contains taxol, an effective anti-cancer drug (Kusari, Singh & Jayabaskaran, 2014). Yew is a shade-tolerant species that grows in vegetated mixed forests in mountainous regions (Kondo, 2016). Yew seedlings grow in understory under low light conditions (Perrin & Mitchell, 2013). When yew seedlings are exposed to full sunlight, photoinhibition occurs (Robakowski & Wyka, 2009). Yew seedlings in full sunlight have decreased survival, so cultivated seedlings should be grown in the shade (Iszkuło, 2010). Nevertheless, yew saplings are often found in open areas of full sunlight (Devaney, Whelan & Jansen, 2015; Myking, Vakkari & Skrøppa, 2009). These findings suggest that there must be some photoprotection differences between seedlings and saplings.

Full sun exposure has a deleterious effect on the photosynthetic system of leaves; plants grown in the shade will suffer more serious photodamage when suddenly exposed to full sun conditions (Demmig-Adams & Adams, 2003). However, over the course of evolution, plants have developed a series of photoprotective mechanisms to avoid photodamage induced by excess light energy (Liu et al., 2020; Meng et al., 2021). For example, photorespiration removes excess electrons and maintains the oxidization of the electron transport chain, thus protecting the photosynthetic apparatus (Busch, 2020). A recent study reported that photorespiration protects photosystem II (PSII) against photooxidative damage by altering electron transport in PSII (Guilherme et al., 2019). However, few studies are focused on photorespiration in yews. Non-photochemical quenching (NPQ) measures excess energy dissipated by heat, which is also important to protecting the photosynthetic apparatus against strong light (Wilk et al., 2013). The main component of NPQ is the rapidly relaxing component (qE), which is associated with trans-thylakoid $\Delta$pH and xanthophyll pigment (violaxanthin, V; antheraxanthin, A; zeaxanthin, Z) conversions (Bernardo et al., 2022). Under high light conditions, it is not known how yew seedlings and saplings will differ in terms of heat dissipation and their xanthophyll cycle. Wax buildup on the leaf epidermis is considered an adaptation to environmental changes such as heat, drought, and high irradiance. Many studies have shown that epicuticular wax plays a role in plant protection under high temperature and drought stress conditions (Wang et al., 2021; Girón-Ramírez et al., 2021; Srinivasan et al., 2008). In addition, a previous study has shown that wax thickness is associated with leaf age (Hauke & Schreiber, 1998). Mohammadian, Watling & Hill (2007) reported that wax on leaf surfaces has a considerable impact on light reflectance and could prevent photoinhibition in *Leucadendron lanigerum*.

In this article, we aimed to explore the adaptability of the Japanese yew to light intensity. In addition, we investigated differences in photosynthetic capacity, photorespiration, and the xanthophyll cycle between yew saplings and seedlings. We also studied the effect of epicuticular wax on photoprotection to reveal potential age-related responses to light availability in the Japanese yew.

## Materials and Methods

### Plant material

This experiment was performed at a nursery at the Northeast Forestry University (45°72′N, 126°63′E) on July 2020. Japanese yews were planted in the nursery for the purposes of this study. We designed a comparative experiment between two groups: (1) the Japanese yew seedling group and (2) the Japanese yew sapling group. Each group included 10 samples. Yew seedlings and saplings were classified by plant height according to the definitions provided in Devaney’s study (Devaney, Whelan & Jansen, 2015): yew seedlings, approximately 0–0.5 m; yew saplings, 0.51–2 m. Three-year-old yew seedlings and 11-year-old yew saplings were selected for the study. The average height, crown width, and base diameter of the seedings were 0.25 m, 27 cm, and 1.26 cm, respectively; the average height, crown width, and base diameter of the saplings were 1.55 m, 96 cm, and 4.16 cm, respectively. Seedlings were planted in plastic pots that were 25 cm in diameter and 20 cm in height (25 × 20 cm), and saplings were planted in plastic pots that were 60 cm in diameter and 55 cm in height (60 × 55 cm). All seedlings and saplings were grown from seed and cultivated under shade cloths situated 2 m above the ground. Shaded plants were grown under a maximum photon flux density (PFD) of about 700 μmol·m^−2^·s^−1^ (40% full sun). Light intensity was measured using a quantum radiometer (LI-189; LI-COR, Lincoln, NE, USA). Plants were watered every 5 days. In the spring, five grams of compound fertilizer was applied to each seeding and ten grams of compound fertilizer was applied to each sapling. Current-year shoots of both seedling and sapling plants were chosen from the mid-crown of each plant to measure pigments, photosynthetic rate, chlorophyll fluorescence, and wax content.

### Measurement of pigments

The chlorophyll (Chl) content and total carotenoids (Car) in the leaves were extracted using 80% acetone. Calcium carbonate was added to prevent the pheophytinization of Chl a. The extracts were filtered through filter paper and analyzed with a UV-2800 system (Hitachi, Shinagawa-ku, Japan). A spectrophotometer was used to estimate chlorophyll content and total carotenoids using extinction coefficients and equations previously described by Lichtenthaler (1987). Xanthophyll cycle components, including violaxanthin (V), antheraxanthin (A) and zeaxanthin (Z), were measured according to Magney et al. (2017). Plants were illuminated at 1,200 μmol·m^−2^·s^−1^ (PFD) for 2 h with a blue-red (8:1) light-emitting diode (LED) after a night of dark-adaptation. Then, the leaf samples were rapidly frozen in liquid nitrogen and extracted with 85% acetone. The quantification of pigments was determined by high-performance liquid chromatography (HPLC) with a Waters 600 solvent pump, a Waters 2,996 diode array detector, a Waters 717 autosampler (Waters Spa, Milford, MA, USA) set to record at 445 nm, and a YMC Carotenoid C30 reverse phase column (5 μm particle size, 250 × 4.6 mm I.D.; YMC America, Inc., Allentown, PA, USA).

### Measurement of the photosynthetic rate

The net photosynthetic rate (Pn), stomatal conductance (Gs), and transpiration rate (Tr) were measured using a LI-6400 portable photosynthesis system (LI-COR, Lincoln, Nebraska, USA). The photosynthetic rate (Pn) response curves to PFD were determined according to outdoor temperature and CO_2_ atmospheric concentration using a coniferous leaf chamber at 30 °C and 380 μmol·mol^−1^ CO_2_ to obtain the light-saturation point (LSP) and light-compensation point (LCP). PFDs were fixed in a sequence of: 1,800; 1,500; 1,200; 1,000; 800; 400; 200; 100; 50; and 0 μmol·m^−2^·s^−1^.

Photorespiration (Pr) was calculated with the following formula:

$\rm {Pr} = {Pn_{O2}^1-Pn_{O2}^2}$

where Pn_O2_^1^ is the photosynthetic rate measured at 21% O_2_ +350 μmol·mol^−1^CO_2_, and Pn_O2_^2^ is the photosynthetic rate measured at 2% O_2_ +350 μmol·mol^−1^CO_2_.

### Measurement of chlorophyll fluorescence parameters

To examine the response of PSII in seedlings and saplings to high light conditions, chlorophyll (Chl) fluorescence was measured in the laboratory with an FMS-2 pulse-modulated fluorometer (Hansatech, Norfolk, UK). Seedling and sapling plants were exposed to 1,200 μmol·m^−2^·s^−1^ PFD irradiance for 2 h using a plant growth lamp (ECO-014, Guangzhou, China). During the 2 h of high light exposure, the maximum quantum yield of photosystem II (Fv/Fm), actual PSII efficiency ( $\Phi$PSII), non-photochemical quenching (NPQ), and high-energy fluorescence quenching (qE) were measured one time, at 30 min intervals. Fv/Fm recovery in darkness for 1.5 h followed the high light exposure. Before measuring Fv/Fm, the sample leaf was dark-adapted for 35 min. Fv/Fm, $\Phi$PSII, NPQ, and qE measurements were taken following the methods described by Johnson et al. (1993).

### Wax determination and removal

To quantify the total weight of the wax on the adaxial and abaxial Japanese yew leaf surfaces, 2 g of leaves were submerged in 30 mL of hexane for 30 s and then rinsed with 2 mL of hexane. Wax extracts (hexane) were then evaporated using N2 gas. The total weight of the wax was expressed based on leaf area, which was determined using a photo image system (LA-S, Hangzhou, China). To identify the impact of epicuticular wax on photosynthetic protection, the wax from the adaxial surface of Japanese yew leaves were removed by gently pressing Blu-Tack (Bostik, Stafford, UK) against the leaf surface a number of times, without causing damage to the leaf surface, according to the methods outlined by Mohammadian, Watling & Hill (2007).

### Statistical analysis

Excel 2022 was used for data input and storage, and the mean values in the table and the figures represent 3–5 replications. All statistical analyses were performed using SPSS statistical software (IBM SPSS Statistics 19.0; Chicago, IL, USA). Student’s t-test was used to analyze the differences between the seedlings and saplings. The experimental data were also subjected to a homogeneity of variance test and a normality test. All charts in this article were plotted in the Origin 9.0 software.

## Results

### Effect of sunlight on appearance of seedling and sapling leaves

**Figure 1:**
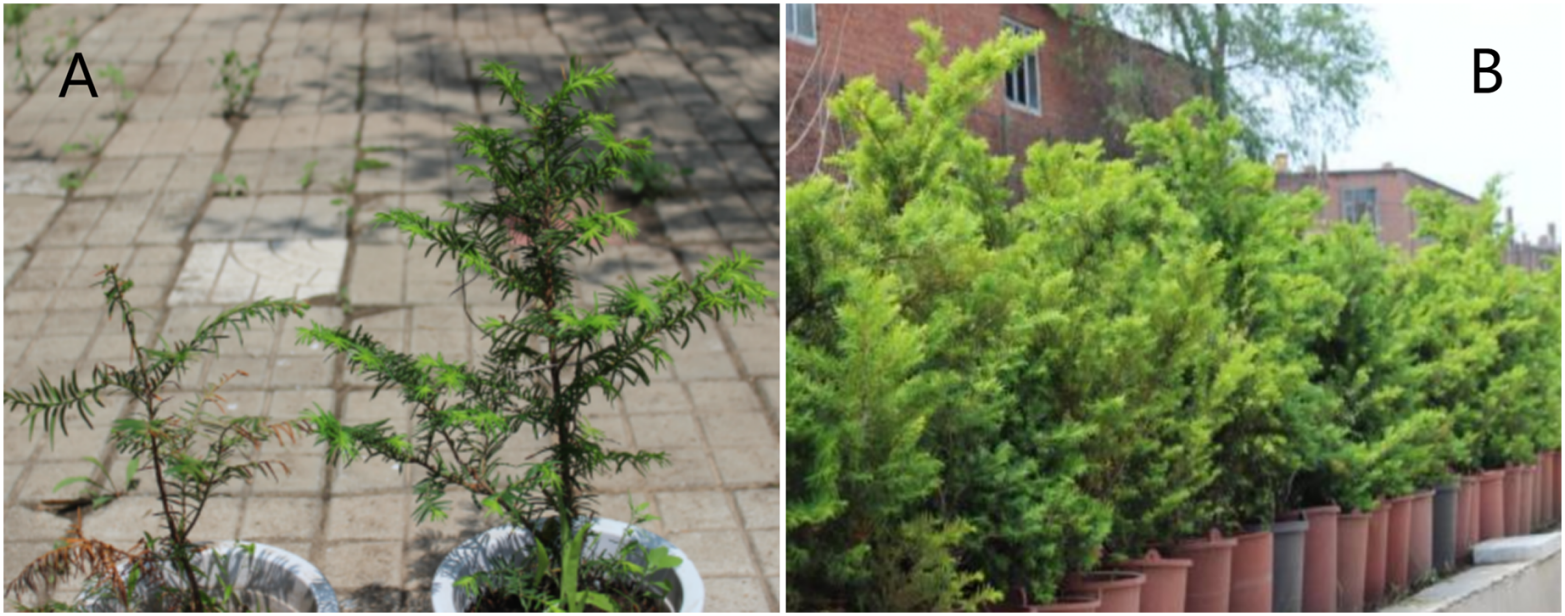
Appearance of seedling and sapling leaves of *Taxus cuspidata* grown under natural light for 90 days. (A) Appearance of 3-year-old Japanese yew seedling leaves. The seedling on the left side of the photograph was exposed to natural light for 90 days. The seedling on the right side of the photograph was grown under shade cloth. (B) Appearance of 11-year-old Japanese yew sapling leaves exposed to natural light for 90 days. Photo credit: Wei Li.

When Japanese yew seedlings were exposed to high light conditions, their foliage turned a bronze color (the left side of Fig. 1A); however, Japanese yew saplings live in open environments (Fig. 1B). Photodamage occurred in seedling leaves exposed to high light conditions, indicating there may be some differences in photoprotection between Japanese yew seedlings and saplings.

### Pigment content

Seedlings and saplings grown under the same light conditions did not show significant differences in Chl b and Chl (a+b; Table 1, *P* > 0.05). Chl a, Car, the Chl a/b ratio, and the Car/Chl ratio in sapling leaves were significantly higher than in seedling leaves (Table 1, *P* < 0.05).

**Table 1:** Changes in Chl a, Chl b, Chl (a+b), and Car contents in Japanese yew seedling and sapling leaves.

Parameter	Seedling leaves	Sapling leaves
Chl a (mg m^−2^)	262.8 ± 6.3a	293.1 ± 7.2b
Chl b (mg m^−2^)	98.2 ± 4.9a	92.9 ± 6.1a
Chl (a+b) (mg m^−2^)	361.1 ± 11.2a	387.3 ± 13.3a
Cars (mg m^−2^)	78.65 ± 4.3a	119.84 ± 5.6b
Chla/b	2.71 ± 0.09a	3.08 ± 0.11b
Cars/Chl	0.22 ± 0.07a	0.31 ± 0.09b

**Note:**

Data are presented as mean ± SE (*n* = 3). Data followed by different lowercase letters in line indicate significance at *P* < 0.05.

### Photosynthesis and photorespiration

**Figure 2:**
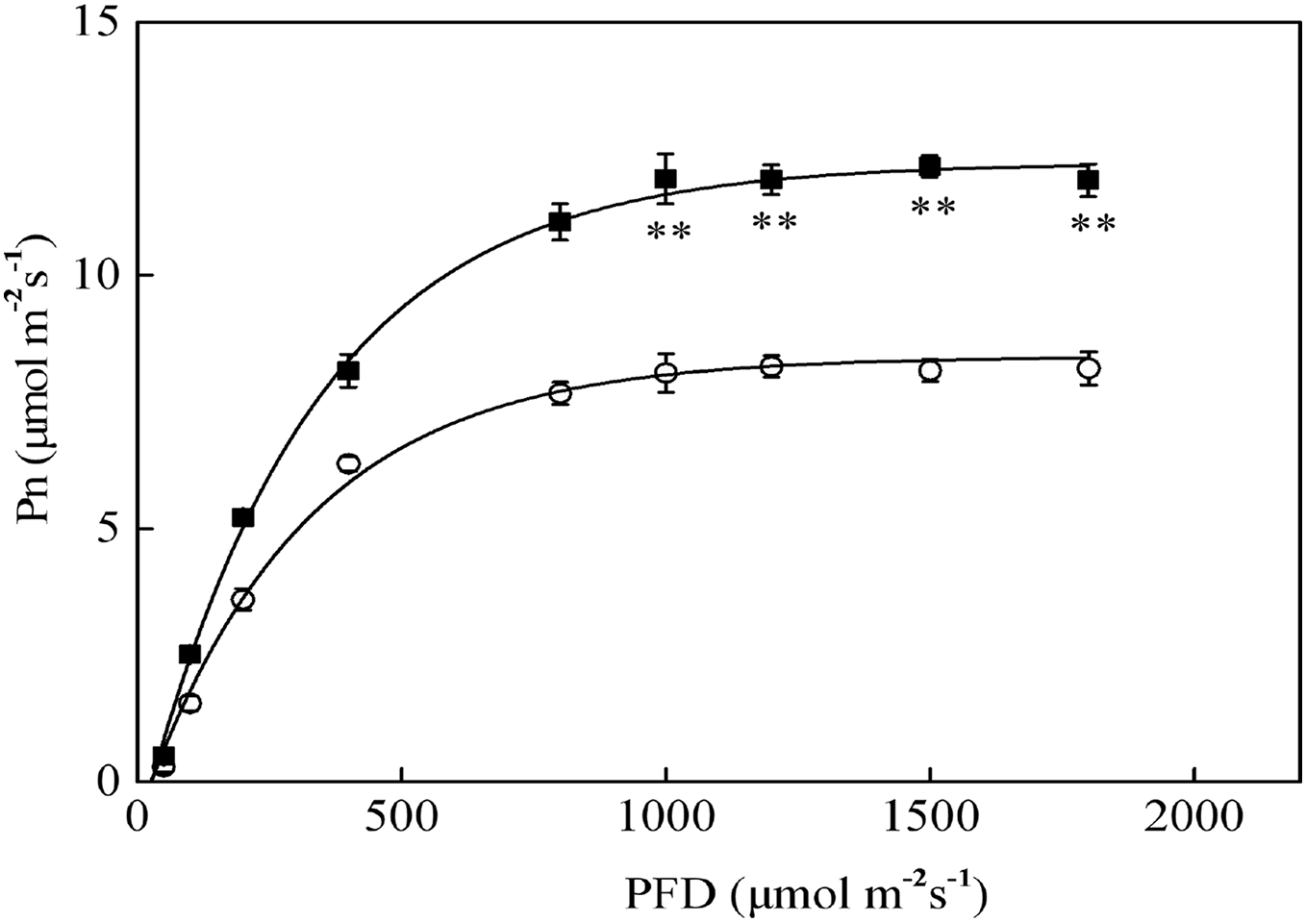
The photosynthesis–photosynthetic photon flux density (Pn-PFD) curves for sapling and seedling leaves. ■ and ○ represent sapling leaves and seedling leaves, respectively. Values are presented as mean ± SE (*n* = 5). Asterisks (**) represent a significant difference, at *P* < 0.01, between sapling and seedling leaves.

The Pn-PFD curves are shown in Fig. 2. The light saturation points (LSP) in sapling and seedling leaves were approximately 1,000 μmol·m^−2^·s^−1^ and 800 μmol·m^−2^·s^−1^ PFD, respectively. The maximum photosynthetic rates (Pn-max) under LSP were approximately 12.1 μmol·m^−2^·s^−1^ in saplings and 8.2 μmol·m^−2^·s^−1^ in seedlings. The sapling leaves had a significantly higher CO_2_ assimilation capacity beyond LSP than the seedling leaves (Fig. 2, *P* < 0.01). As shown in Fig. 2, 1,200 μmol·m^−2^·s^−1^ PFD is a strong light condition for the Japanese yew.

**Figure 3:**
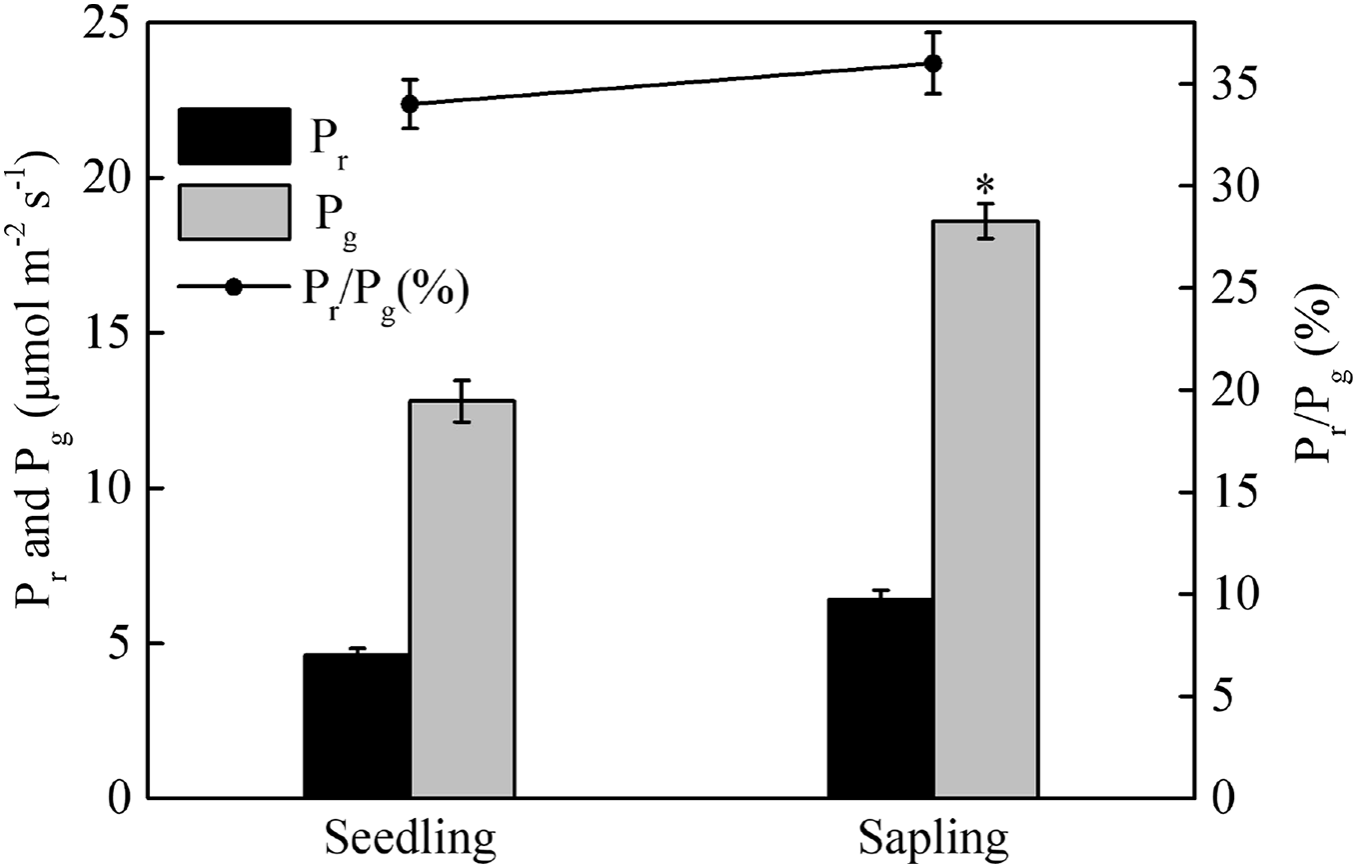
Photorespiration (Pr), gross photosynthetic rate (Pg), and the ratio of photorespiration to gross photosynthetic rate (Pr/Pg) in sapling and seedling leaves. Values are presented as mean ± SE (*n* = 3). An asterisk (*) represents a significant difference, at *P* < 0.05, between sapling and seedling leaves.

Compared with sapling leaves, photorespiration (Pr), gross photosynthetic rate (Pg), and the Pr/Pg ratio were all lower in seedling leaves, but only the difference in Pg between sapling and seedling leaves reached statistical significance (Fig. 3, *P* > 0.05).

### The maximum quantum yield of photosystem II

**Figure 4:**
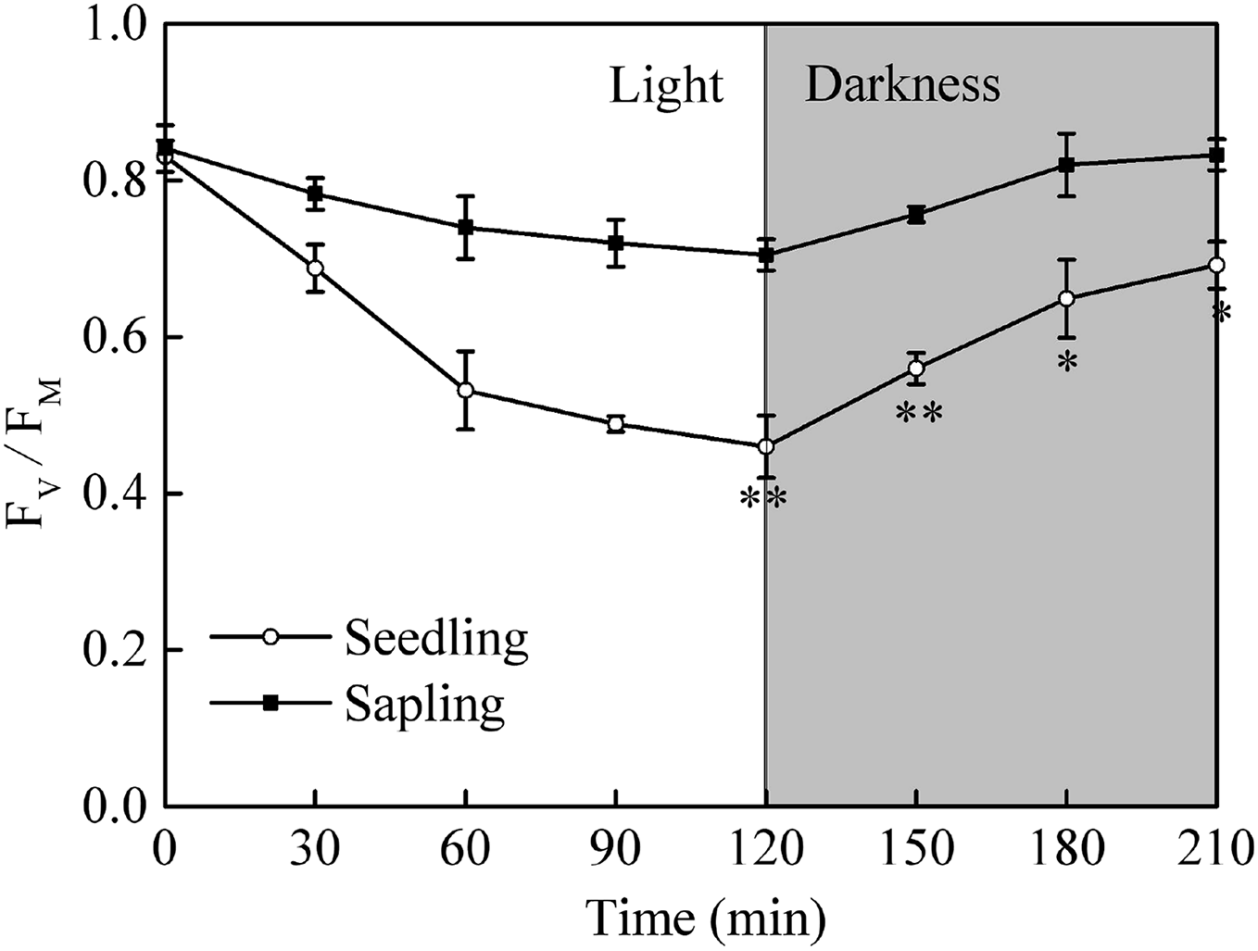
The change in Fv/Fm in sapling and seedling leaves under 1,200 μmol·m^−2^·s^−1^ PFD for 120 min and recovery under dark conditions for 90 min. ■ and ○ represent sapling leaves and seedling leaves, respectively. Values are presented as mean ± SE (*n* = 5). Asterisks (* and **) represent significant differences, at *P* < 0.05 and *P* < 0.01, respectively, between sapling and seedling leaves.

After a period of dark adaptation, the maximum quantum yields of photosystem II (Fv/Fm) in seedling and sapling leaves were 0.83 and 0.84, respectively (Fig. 4, *P* > 0.05). Under high light conditions, the Fv/Fm in both seedling and sapling leaves decreased. However, a more significant decrease in Fv/Fm was observed in seedling leaves at 120 min (Fig. 4, *P* < 0.01), indicating that seedling leaves were more susceptible to high light conditions than sapling leaves. When the plants were returned to dark conditions, Fv/Fm recovery in seedling leaves was significantly slower than in sapling leaves (Fig. 4, *P* < 0.05). After 90 m of darkness, the Fv/Fm was still not completely restored in seedling leaves.

### Chlorophyll fluorescence changes under high light conditions

**Figure 5:**
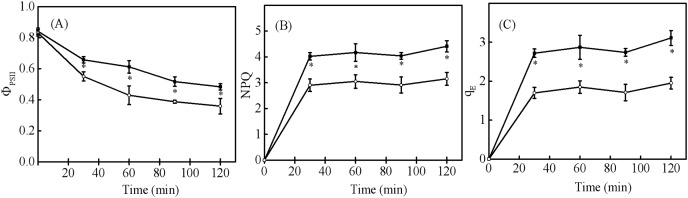
Changes in (A) actual photosystem II efficiency ( $\Phi$PSII), (B) non-photochemical quenching (NPQ), and (C) high-energy fluorescence quenching (qE) in sapling and seedling leaves under 1,200 μmol·m^−2^·s^−1^ PFD. ■ and ○ represent sapling leaves and seedling leaves, respectively. Values are presented as mean ± SE (*n* = 4). An asterisk (*) represents a significant difference, at *P* < 0.05, between sapling and seedling leaves.

When seedling and sapling leaves were exposed to high light conditions, there was a pronounced decline of $\Phi$PSII observed over time (Fig. 5A). However, $\Phi$PSII in sapling leaves was higher than in seedling leaves (*P* < 0.05). NPQ and qE in sapling leaves also remained higher than in seedling leaves (Figs. 5B and 5C, *P* < 0.05).

### Change in xanthophyll cycle pigments

**Figure 6:**
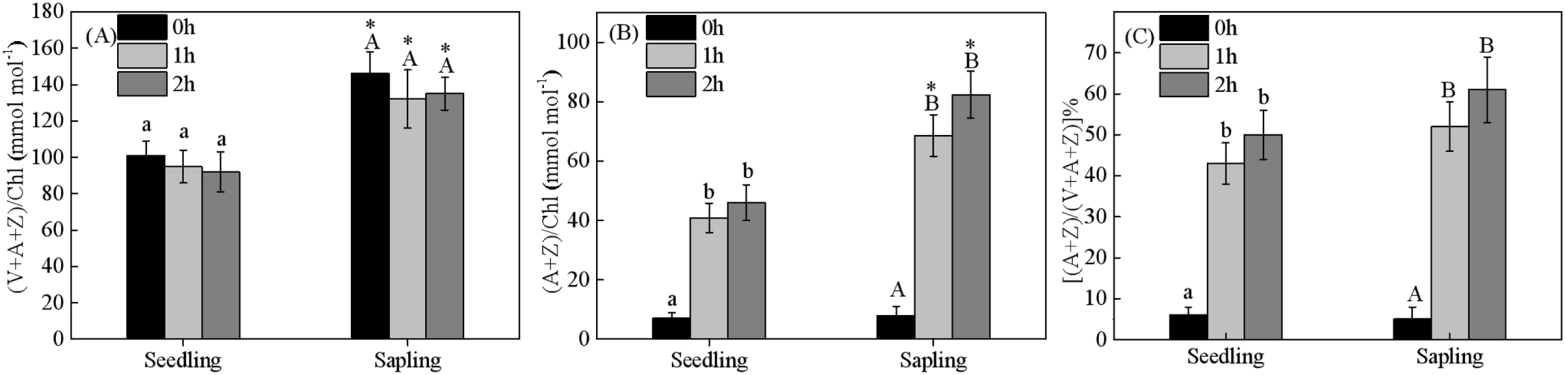
Changes in (A) xanthophyll cycle pigment contents, (B) the contents of de-epoxidation components, and (C) the de-epoxidation state in sapling and seedling leaves. Attached leaves were vertically exposed to 1,200 μmol·m^−2^·s^−1^ PFD for 0, 1, and 2 h. Values of (A) and (B) are expressed based on chlorophyll and presented as mean ± SE (*n* = 3). Different lowercase letters represent significance at *P* < 0.05 in seedling leaves. Different capital letters represent significance at *P* < 0.05 in sapling leaves. An asterisk (*) represents a significant differences, at *P* < 0.05, between sapling and seedling leaves.

Energy dissipation is closely related to xanthophyll cycle pigments. Compared within seedling leaves, the size of the xanthophyll cycle pool was higher in sapling leaves (Figs. 6A and 6B, *P* < 0.05). There were no significant differences in de-epoxidation levels in the components of xanthophyll cycle pigments in the seedling or sapling leaves exposed to 1,200 μmol·m^−2^·s^−1^ PFD for 2 h (Fig. 6C, *P* < 0.05).

### The effect of epicuticular wax on gas-exchange

**Figure 7:**
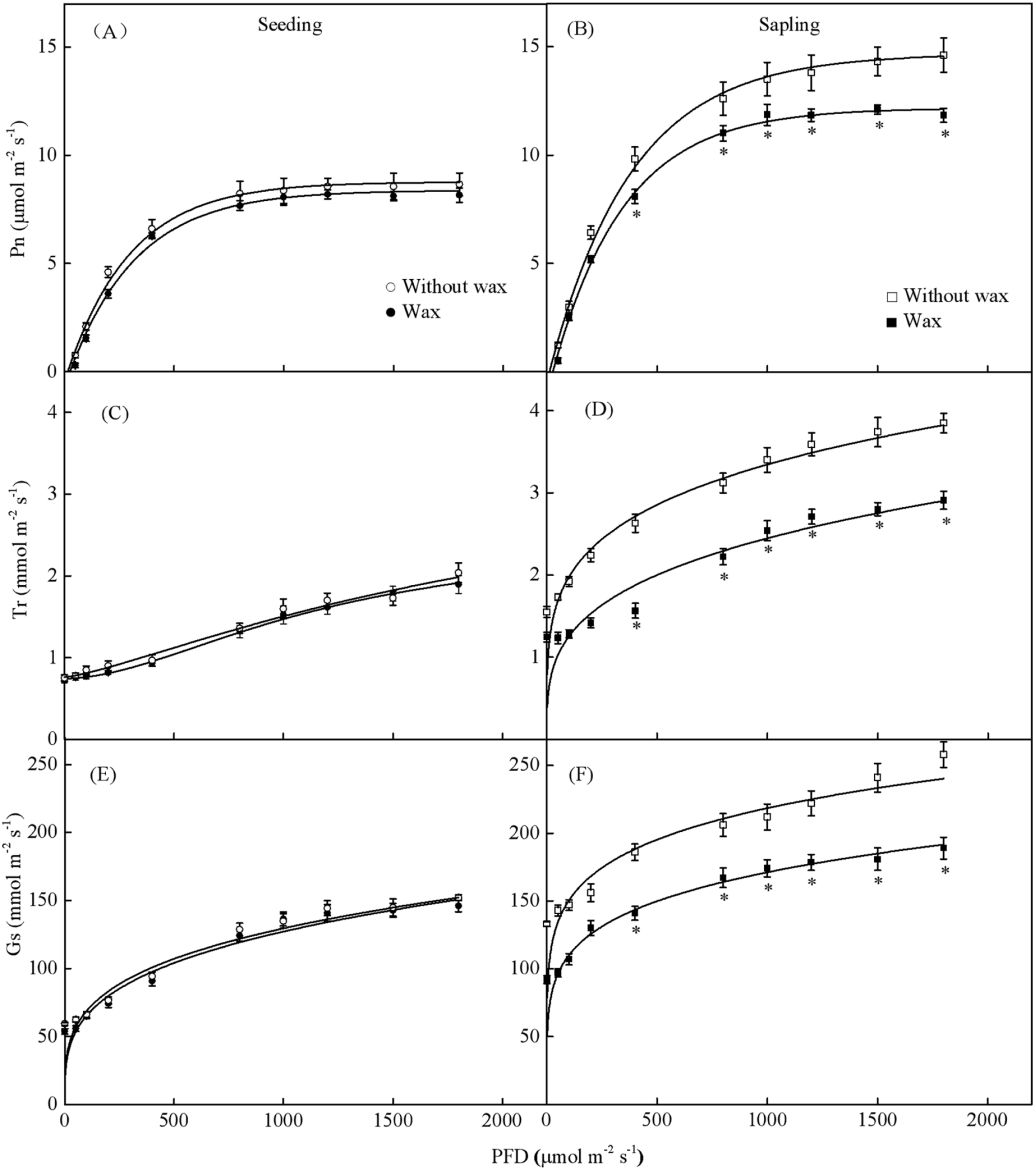
Gas-exchange parameters with PFD for sapling and seedling leaves with and without waxy coverage. (A), (C), and (E) are the photosynthetic rate (Pn), transpiration rate (Tr), and stomatal conductance (Gs) in seedling leaves, respectively. (B), (D), and (F) are the Pn, Tr, and Gs in sapling leaves, respectively. A solid marking represents the presence of wax, and an open marking represents no wax present. Values are presented as mean ± SE (*n* = 5). An asterisk (*) represents a significant difference, at *P* < 0.05, between sapling and seedling leaves.

When treated with 500 PFD to 2,000 PFD, the photosynthetic rate in sapling leaves without wax was significantly higher than in leaves with wax (Fig. 7B, *P* < 0.05). However, there was no significant difference in the photosynthetic rate in the seedling leaves with or without wax (Fig. 7A, *P* > 0.05). The sapling leaves without wax also had higher Gs and Tr values compared to the sapling leaves with wax (Figs. 7D and 7F, *P* < 0.05). However, there was no significant difference in Gs and Tr values in the seedling leaves with or without wax (Figs. 7C and 7E, *P* > 0.05).

**Figure 8:**
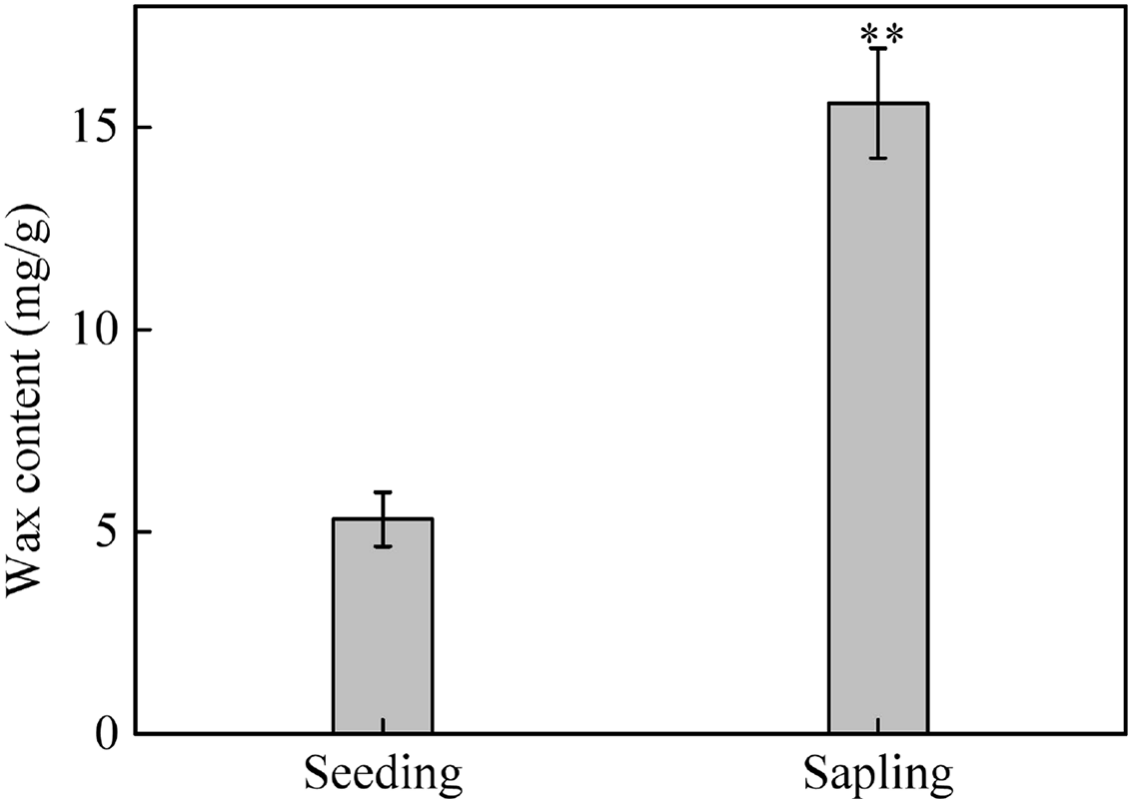
The content of epicuticular wax in sapling and seedling leaves. Values are presented as mean ± SE (*n* = 4). Asterisks (**) represent a significant difference, at *P* < 0.05, between sapling and seedling leaves.

The total wax weight was significantly higher on sapling leaves than on seedling leaves (Fig. 8, *P* < 0.01).

### Chlorophyll fluorescence with or without wax

**Figure 9:**
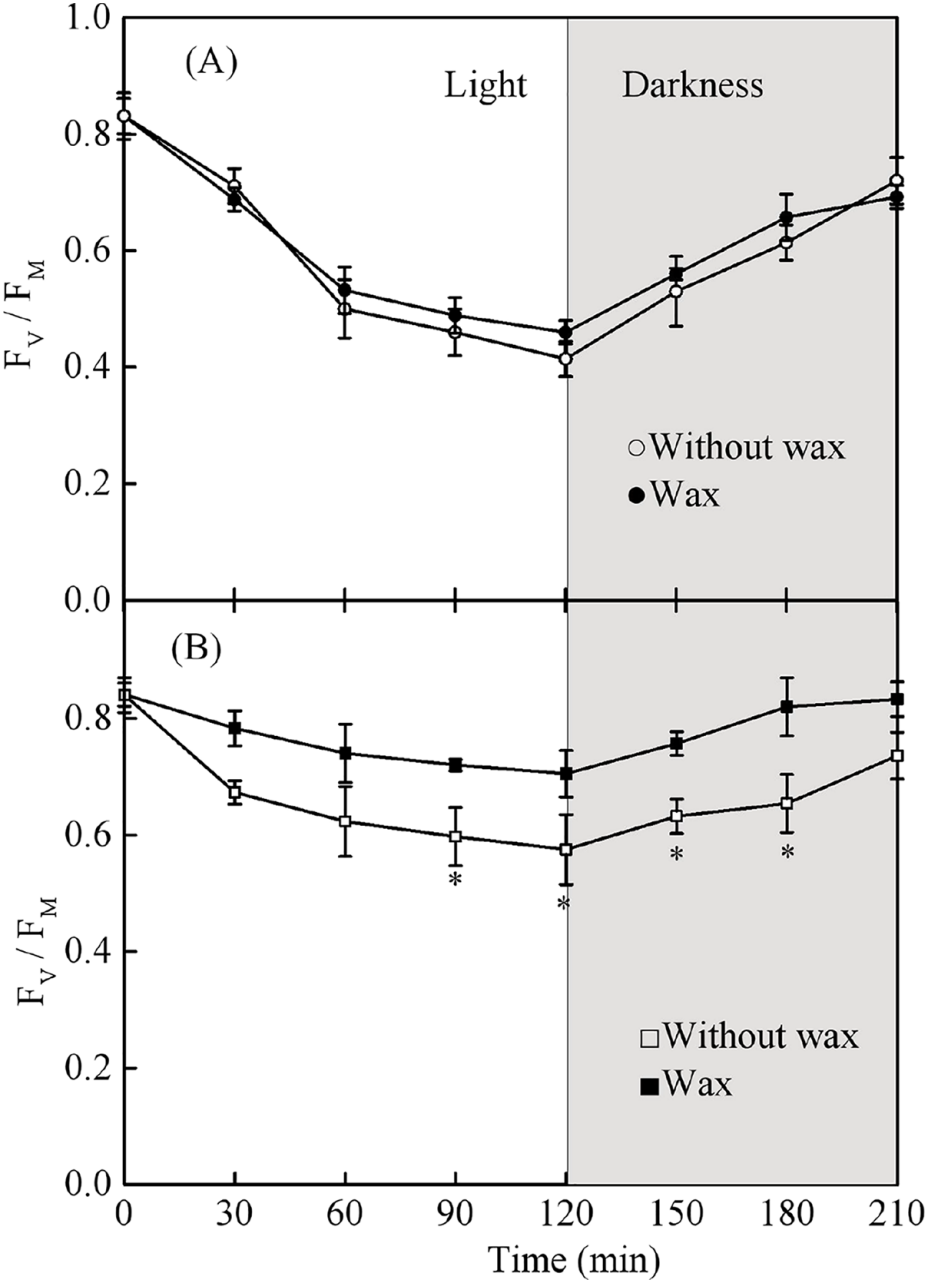
(A) The change in Fv/Fm in the adaxial surface of seedling leaves with and without waxy coverage. (B) The change in Fv/Fm in the adaxial surface of sapling leaves with and without waxy coverage. A solid marking represents the presence of wax, and an open marking represents no wax present. Values are presented as mean ± SE (*n* = 5). An asterisk (*) represents a significant difference, at *P* < 0.05, between leaves with wax and leaves without wax.

Leaves with and without wax showed a reduction in Fv/Fm when grown under full sun for 2 h (Fig. 9). Fv/Fm values were restored to pre-exposure values after returning to low light conditions for 1.5 h. The Fv/Fm in sapling leaves with wax was significantly higher than in sapling leaves without wax after exposure to full sun for 2 h (Fig. 9B, *P* < 0.05). However, there was no significant difference in Fv/Fm in the seedling leaves with or without wax (Fig. 9A, *P* > 0.05).

## Discussion

The chlorophyll content, Chl a/Chl b ratio, and Pn were all higher in sapling leaves than in seedling leaves (Table 1; Fig. 2), indicating that sapling leaves had developed a photosynthetic process where more excitation energy is utilized for CO_2_ assimilation rather than heat dissipation. The Fv/Fm was similar at the initial stage of full sun conditions in both seedling and sampling leaves. Fv/Fm represents the original activity of PSII (Zhang et al., 2020), indicating that the activity of PSII was not the limiting factor for photosynthesis in seedling leaves (Fig. 4).

Plants have developed several photoprotective mechanisms to prevent photodamage induced by high irradiance (Zhao et al., 2017; Guha et al., 2022; Son et al., 2021). Photorespiration plays a key role in dissipating excess photochemical energy in C3 plants (Lim et al., 2020). Pr/Pg represents the proportion of excitation energy allocated to photorespiration. However, there was no significant difference in Pr and ratio of Pr/Pg between seedlings and saplings (Fig. 3), indicating that photorespiration did not play the key role in mitigating deleterious effects of high light conditions in the Japanese yew.

A lower ΦPSII was observed in seedling leaves under controlled light (Fig. 5A), indicating that the light energy distributed to the photochemical reaction in seedling leaves was lower than that in sapling leaves, and that more excessive energy was produced in seedling leaves. However, the lower qE in seedling leaves indicated that less excessive energy was dissipated by heat in seedling leaves than in sapling leaves (Fig. 5C). Previous studies reported that qE is promoted by violaxanthin (V) that is converted to antheraxanthin (A) and zeaxanthin (Z) *via* enzyme-catalyzed de-epoxidations (Magney et al., 2017). After 2 h of exposure to high light conditions, there was an increase in the de-epoxidation components of xanthophyll pigments observed in sapling leaves compared to seedling leaves (Fig. 6), which was supported by the observed changes in qE. Compared with sapling leaves, there were less xanthophyll pigments per Chl a in the seedling leaves making them unsuitable for the high light environment as they could not cope with the excess energy.

According to previous studies, wax content on leaves prevents water from entering the pores and increases the resistance of gas diffusion (Mohammadian, Watling & Hill, 2007). Because stomata are partially occluded by wax, decreasing the cross-sectional area for gas diffusion, the stomatal conductance of leaves should also decrease when wax is present. In our study, the increase of Pn and Tr observed in sapling leaves without wax was likely the result of an increase in the stomatal conductance of the leaves (Figs. 7A and 7B). However, Pn and Tr in seedling leaves did not change significantly after wax removal. This might be due to the significantly lower wax content in seedling leaves compared to sapling leaves (Fig. 8).

Robinson, Lovelock & Osmond (1993) found that wax is a very important protection against photoinhibition in *Cotyledon orbiculate*. Mohammadian, Watling & Hill (2007) reported that the increased reflectance of light by wax on the leaf surface prevents photodamage in the PSII reaction center. In this study, after exposure to high light conditions for 2 h, sapling leaves without wax showed greater photoinhibition than control plants (Fig. 9B). However, there was no significant difference in seedling leaves with or without wax (Fig. 9A), suggesting that wax on sapling leaves played a more important role in photoprotection under high light conditions than it did on seedling leaves. This is likely related to the content of the wax secreted by the seedling and sapling leaves. The amount of wax secretion might also be related to tree age (Fig. 8). Since light reflectance was not measured, further studies are needed to support these results.

## Conclusions

Sapling leaves had a higher photosynthetic rate and thermal dissipation depending on the xanthophyll cycle than seedling leaves. Wax coverage significantly reduced photoinhibition, and the deposition of wax in T. *cuspidata* is likely related to tree age, with the content of wax on seedling leaves much lower than on sapling leaves. The findings of this study indicate that Japanese yew seedlings are more susceptible to high light conditions than Japanese yew saplings. High light conditions may lead to a high mortality for Japanese yew seedlings.

## Supplemental Information

10.7717/peerj.14757/supp-1 Supplemental Information 1 FvFm data.

10.7717/peerj.14757/supp-2 Supplemental Information 2 Light response curve data.

10.7717/peerj.14757/supp-3 Supplemental Information 3 qE data.

10.7717/peerj.14757/supp-4 Supplemental Information 4 psii data.

10.7717/peerj.14757/supp-5 Supplemental Information 5 Lutein cycle data.

10.7717/peerj.14757/supp-6 Supplemental Information 6 NPQ data.

10.7717/peerj.14757/supp-7 Supplemental Information 7 Fv Fm data.

10.7717/peerj.14757/supp-8 Supplemental Information 8 Stomatal conductance data.

10.7717/peerj.14757/supp-9 Supplemental Information 9 Pn data.

10.7717/peerj.14757/supp-10 Supplemental Information 10 Transpiration data.

10.7717/peerj.14757/supp-11 Supplemental Information 11 Wax content data.

10.7717/peerj.14757/supp-12 Supplemental Information 12 Photorespiration/total photosynthesis.

10.7717/peerj.14757/supp-13 Supplemental Information 13 Light fusion rate Pn.

10.7717/peerj.14757/supp-14 Supplemental Information 14 Light intensity PAR.

10.7717/peerj.14757/supp-15 Supplemental Information 15 Raw data. Response of photosynthesis, the xanthophyll cycle, and wax in Japanese yew (*Taxus cuspidata* L.) seedlings and saplings under high light conditions

## Additional Information and Declarations

### Data Availability

The following information was supplied regarding data availability: The raw measurements are available in the Supplemental Files.
